# Stone in the Bladder

**Published:** 1901-06-01

**Authors:** 


### STONE IN THE BLADDER.

Speaking recently at a meeting of the East Sussex Medico-Chirurgical Society, Dr. P. J. Freyer[1] passed in review the generally accepted symptoms of stone in the bladder. Increased frequency of micturition is, he said, the earliest and the most constant symptom of stone. The bladder becomes irritable—frets as it were—at the presence of its unnatural and unwelcome tenant, and this, like most of the symptoms of stone, is aggravated by exercise and diminished by rest. Consequently it is less troublesome at night, when the patient is lying down, than in the day, when he is going about; exactly the reverse of what occurs with enlarged prostate. But increased frequency of micturition occurring by itself, apart from one or more of the other symptoms of stone, is of little diagnostic importance. It may occur with any disease of the urinary tract, and indeed in perfect health under the influence of drugs, certain articles of food, or with the sudden setting-in of cold weather. Again in women, notwithstanding the infrequency with which stone in the bladder is met with among them, two of the symptoms of stone are very common; namely, pain and increased frequency of micturition, symptoms which in the great majority of cases are due to some flexion, enlargement, or inflammatory state of the uterus, causing pressure on the bladder. Vascular caruncle is also a not uncommon cause of these symptoms.

Pain in stone in the bladder is rarely referred to the bladder itself. It is reflex in character, and is situated near the end of the penis, at the posterior aspect of the glans, most frequently in the position of the frenum, setting in and gradually increasing in intensity towards the end of the act of micturition, as the bare walls of the bladder come in contact with the stone. Then as the bladder gradually refills the pain disappears, the urine acting as a buffer between the stone and the bladder walls. The pain is much more intense in children than in aged persons. Jolting from any form of exercise, by which the stone is driven against the bladder wall, will also bring it on. This reflex pain referred to the glans used to be regarded as pathognomonic of vesical calculus; but recent observations have shown that it occurs with other conditions, namely stricture of the urethra, enlarged prostatic middle lobe, tubercle of the prostate, local cystitis, ulcer of the bladder, pendulous tumour falling against the neck of the bladder, clots of blood or thick mucus lying at the inner orifice of the urethra, and also, though more rarely, by stone in the ureter or pelvis of the kidney. In stricture of the urethra the pain accompanies the act of micturition and is felt in the seat of stricture. In prostatic disease the pain precedes the act, and is as a rule referred to the hypogastric region. In coming to a conclusion, as between stone and enlarged prostate, it must be remembered that the two conditions often exist together. When great irritability of the bladder with hæmaturia occurs in a patient with enlarged prostate, the presence of stone should always be suspected.

The hæmaturia which occurs with stone in the bladder presents three characteristics—(*a*) it comes on gradually, sudden and profuse hæmorrhage not being characteristic of stone; (*b*) it generally appears towards the end of the act of micturition, the earlier portion of the stream being clear, the latter tinged with blood and winding up with a few drops of bright clear blood; (*c*) exercise increases the tendency to its occurrence. In tumours of the bladder the bleeding will be more profuse than in cases of stone, and is painless as a rule, the urine being uniformly mixed with blood and containing large, irregular clots, and perhaps some portions of tumour débris. In tuberculous ulcer of the bladder the bleeding is very like that connected with stone; but the co-existence of tubercle in other parts, particularly in the epididymis, vesiculæ seminales and prostate, will help in the diagnosis. But there is no disease the symptoms of which so closely resemble those of stone as tubercle of the bladder. In hæmorrhage from the kidney the urine is as a rule uniformly mixed with blood, is frequently smoky in colour, and often contains dark, worm-like clots—casts of the ureters.

Sudden stoppage of the flow of urine may be caused by other conditions besides stone in the bladder, one of which, mentioned by Dr. Freyer, is the somewhat peculiar one of a prolapsed lower end of a ureter containing a stone and plugging the urethral orifice.

Before giving a definite diagnosis of stone in the bladder, the stone should be felt. First a rectal examination should be made, then the bladder should be sounded, and if that should fail to reveal the stone the aspirator and a large-sized canula, such as is employed in Bigelow's operation, should be used. If a small stone be present it will almost to a certainty click against the tube—and it may perchance pass through the canula without the necessity of crushing it. A stone, however, may be so fixed as to require the use of the cystoscope for its discovery, while when all these methods fail it may be advisable or necessary to open the bladder, preferably suprapubically, so as if possible at the same time to discover and to remove the cause of the symptoms.

[1] Brit. Med. Jour., May 25.

